# Low-SNR and BER reduction in UWOC systems using DESN and CNN-TCN deep learning models

**DOI:** 10.1038/s41598-025-31837-x

**Published:** 2025-12-27

**Authors:** Wessam M. Salama, Moustafa H. Aly, Eman S. Amer

**Affiliations:** 1 Department of Computer Engineering, Faculty of Engineering, Pharos University, Canal El Mahmoudia Street, Beside Green Plaza Complex, 21648 Alexandria, Egypt; 2 Department of Electronics and Communications Engineering, College of Engineering and Technology, Arab Academy for Science, Technology and Marine Transport, 1029, Alexandria, Egypt; 3 Faculty of Industrial and Energy Technology, Borg Al Arab Technological University, Alexandria, Egypt

**Keywords:** Convolutional neural network, Long short-term memory, Wireless optical communication, Pulse amplitude modulation, Recursive least square, Deep echo state network, Engineering, Mathematics and computing

## Abstract

Both commercial and scientific underwater wireless optical communication (UWOC) systems are essential and significant for several applications with the ability to provide high data transmission rates over distances up to tens of meters. There are several research gaps for UWOC like the limitation of the UWOC performance which is impacted by the intrinsic characteristics of ocean water which includes losses that reduce the signal-to-noise ratio (SNR) and impede communication quality. The main objective of this study is to enhance the performance of the UWOC system by utilizing recent artificial intelligence (AI) technologies to reduce the bit error rate (BER) with different neural network (NN) models. We apply both pulse amplitude modulation (PAM) and quadrature phase shift keying-orthogonal frequency division multiplexing (QPSK-OFDM) with several NN models across a range of underwater transmission ranges with PAM of 100, 125, 167 m and QPSK-OFDM of 100, 110, 120, 130 m. Deep echo state network (DESN) and recursive least square (RLS) are the two channel estimation techniques used. A variety of deep learning (DL) models and convolutional neural network (CNN) are assessed to improve system robustness and efficiency. CNN-RNN-AM shows performance gains of 18.9, 23.5, 33.3, and 29.4% at 100, 110, 120, and 130 m, respectively. CNN-LSTM-AM shows gains ranging from 10 to 17.6%; and TCN-LSTM-AM shows the largest gains, 29.4, 41.17, 53.3, and 71.4%, at the same distances. As implications of these results that, by comparison, DESN improves by up to 25% at 167 m, while PAM with TCN-LSTM-AM only improves by 17.3% at 100 m utilizing RLS. The most notable improvements are seen in CNN-LSTM-AM and TCN-LSTM-AM, which achieve accuracy gains of + 10.41 and + 11.80%, respectively, along with notable decreases in error metrics like mean squared error (MSE) of 58.46 and 66.15%, root mean square error (RMSE) of 30.48 and 41.90%, and mean absolute error (MAE) of 34.94 and 40.96%. Remarkably, TCN-RNN-AM exhibits a significant improvement in accuracy of 7.25% and RMSE of 32.38%, but it also experiences a minor rise in MAE of −4.82%, suggesting less consistent absolute error handling. When AM is added to CNN-LSTM, the accuracy increases from 94.33 to 96.77%, and the MSE, RMSE, and MAE decrease to 0.0054, 0.073, and 0.054, respectively. The TCN-LSTM model with AM also attains the maximum accuracy of 97.99%, indicating a 3.65% improvement. These findings demonstrate how well DL works in conjunction with sophisticated modulation and channel estimation methods to significantly enhance UWOC system performance in demanding underwater conditions.

## Introduction

There are several techniques in underwater communication systems to link the distributed nodes in an UWOC system, Underwater Acoustic Communication (UWAC), and Underwater Radio Frequency (RF) Communication (URFC)^1^. These techniques are used to deliver data surrounding each node and provide underwater service for the applications of scientific and commercial, such as water quality monitoring. The main advantage of using UWOC is that its bandwidth of the light is in the range of hundreds of terahertz. Despite the advantages of UWOC such as high data rates, high security, and low latency communication, there are some drawbacks in these UWOC systems. In brief, underwater optical channels interface substantial interference caused by absorption and scattering phenomena. Both scattering and absorption decrease the achievable link length of the UWOC systems.

By providing strong tools for feature extraction, noise reduction, and complicated pattern identification, DL model^2^ has completely transformed signal processing. These skills are particularly useful in demanding settings like UWOC systems. Recent studies^3–5^ have investigated sophisticated modulation techniques that enhance spectral efficiency and signal robustness, such as PAM and QPSK-OFDM. Furthermore, adaptive signal processing techniques like RLS have been used for real-time channel equalization.

In this regard, DL models have demonstrated considerable promise in modeling underwater optical channels, particularly those that combine CNNs^6^, RNNs^7^, LSTM^8^ networks, AM, and CNN^9^. Better channel estimates, signal amplification, and overall system robustness result from these designs’ capacity to learn both spatial and temporal information.

By using a suite of DL models to enhance the datasets obtained from the work in Ref.^10^, this paper primarily contributes to the improvement of UWOC systems performance. Using raw signal data as a baseline, K. Wang et al.^10^. estimated the channel using modulation schemes like QPSK-OFDM and PAM for different underwater distances (e.g., 100, 110, 120, 130 m and 100, 125, 167 m) and signal processing techniques like RLS and DESN. The simulations used in the work are based on these MATLAB-generated datasets, which show the link between received power and BER under various circumstances. Building on this foundation, we propose a comparative analysis of several advanced DL architectures, including CNN-RNN, CNN-AM, CNN-RNN-AM, CNN-LSTM, CNN-LSTM-AM, TCN, TCN-RNN, TCN-AM, TCN-RNN-AM, TCN-LSTM, and TCN-LSTM-AM, to improve system robustness under low SNR conditions and reduce noise and distortion. Both PAM and QAM-OFDM signals are considered in the evaluation.

TCN is further introduced in this research as a crucial part of the suggested DL architecture for UWOC signal recovery. Long-term temporal dependencies can be effectively modeled while maintaining sequence order thanks to TCNs causal and dilated convolutions. In comparison to conventional RNNs, they also provide faster training, and enhanced resilience to vanishing gradient problems.

### Research problem

Systems for UWOC provide minimal latency and high data rates for a range of maritime applications. However, channel impairments such noise, turbulence, scattering, and absorption significantly limit their performance, especially when operating at low SNR levels or over long distances. These issues are only partially addressed by conventional signal processing algorithms (like RLS and DESN) and modulation techniques (like QPSK-OFDM and PAM). A reliable, flexible, and scalable method is still desperately needed to enhance signal quality and lower BER in practical UWOC settings.

### Research motivation

Enhancing UWOC system resilience is crucial as environmental monitoring, and ocean exploration depends more on dependable communication. The ability of DL to predict nonlinearities and uncover hidden characteristics in intricate datasets has shown promise. There is a chance to greatly improve signal recovery, lower BER, and get beyond the drawbacks of conventional techniques by utilizing DL architectures, particularly TCN, RNNs, LSTM and AM. The goal of this paper is to use real-world datasets to fully utilize these model’s potential to increase the precision, resilience, and effectiveness of UWOC systems.

### Research objectives

- Make use of and expand on current UWOC datasets in Ref^10^. that include measurements of received power and BER under different channel conditions and modulation methods.
- Create and contrast a variety of DL models with and without AM.
- Improve UWOC performance by employing sophisticated DL frameworks to lower BER and increase tolerance to low-SNR signals.
- Determine the best model architecture for UWOC signal recovery by assessing model performance using important metrics as accuracy, MSE, RMSE, and MAE.

### Research contribution

In order to improve signal processing and channel estimation, this paper applies DL models-based upgrades to the datasets in Ref.^10^, which contain real-world UWOC measurements. The study presents and assesses hybrid DL models designed for UWOC, showcasing their potential to more accurately capture both spatial and temporal data. For simulating underwater signal dynamics, we suggest TCN network, an innovative and efficient architecture that outperforms conventional models in terms of accuracy and computing efficiency. The models undergo a thorough evaluation using a variety of performance indicators. The outcomes show notable gains in BER and other important metrics, particularly when the TCN-LSTM-AM design is used. Through appropriate regularization and architectural integration, TCN-LSTM-AM, overfitting and training complexity can be addressed.

The remainder of the paper is structured as follows: The related work is introduced in Sect. 2. The methodologies used in the paper are listed in Sect. 3. Results and analysis based on simulation and assessment parameters are displayed and explained in Sect. 4. The findings are concluded in Sect. 5, which also lists suggestions for future work.

## Related work

In order to achieve high data rate performance under a variety of underwater environments, UWOC systems have substantial developments and methodological diversity. Different trade-offs between data rate and transmission distance have been shown in empirical research. For example, Jiang, R. et al.^8^ achieved a 138 m transmission at 1 Mbps by using a high-power optical source. A single-photon avalanche diode was used by Yuan, J. et al., which produced transmission lengths of 117 m at 2 Mbps and 144 m at 500 bps. Zandi, I. et al.^11^ used a laser-based white-light source, to expand these efforts, achieving 8.7 Gbps at 2.3 m and 560 Mbps at 90 m. Many channel equalization strategies have been used to reduce distortions caused by the channel. The Least Mean Squares (LMS) technique was used by Abd-Elgawad, L.A. et al.^12^, Linear Minimum Mean Square Error (LMMSE) estimate was used bt Grisales-Noreña, L.F. et al.^13^, and RLS was used by Grebien, S. et al.^14^ and Gupta, S.K. et al.^15^.

Several applied models have been used in previous works for improving different systems. G. Singh et al.^16^ presented CNN-RNN based hybrid DL for predicting fluctuations in the stock market. Furthermore, M. Zuo et al.^17^, used CNN-AM to improve incorporating AM. P. Tripathi et al.^18^ interested in CNN-RNN-AM to develop automatic modulation classification on resource-constrained end devices. Development of a CNN-LSTM used DL for motor imagery EEG classification for brain computer interface applications^19^. TCN-RNN approach used in chaotic time series prediction in^20^. TCN-LSTM was presented by X. Wang et al.^21^ to enhance temperature prediction model for rotary kilns and by X. Xu et al.^22^ in short-term power load forecasting.

## Methodology

The purpose of this study is to improve the performance of UWOC systems by adopting and evaluating several DL architectures. Because of their complementing abilities to handle spatial-temporal aspects, sequential dependencies, and dynamic signal characteristics, CNN-LSTM-AM, CNN-RNN-AM, and TCN-LSTM-AM are chosen for further examination among the tested models. Convolutional layers are used in the CNN-LSTM-AM model to efficiently extract spatial characteristics from optical data. Long-term temporal dependencies are then captured by LSTM units. By using AMs to dynamically focus on pertinent signal components, the CNN-RNN-AM model enhances interpretability and resilience in underwater environments that fluctuate.

By combining dilated causal convolutions from TCNs with LSTM and attention layers, the TCN-LSTM-AM architecture allows for better resistance to vanishing gradients, more comprehensive temporal context modeling, and effective parallel processing. To ascertain whether these models are appropriate for real-world UWOC applications, they are trained and evaluated on improved datasets taken from^10^. Their performance is evaluated using BER, MSE, RMSE, and MAE.

These models offer a comprehensive approach to address the difficulties associated with underwater communication by combining the capabilities of convolutional layers for spatial feature extraction such as edges, corners, textures, and shapes, LSTM networks for temporal dependencies, and AMs for dynamic focus. It is a useful tool for developing underwater communication technologies because of its ability to predict BER accurately and recommend mitigation strategies, which greatly improves the dependability and effectiveness of UWOC systems. This is performed in four steps, first is preprocessing to input dataset, then feature extraction by CNN or TCN, after that using several proposed models CNN-LSTM, CNN-RNN-AM and TCN-LSTM-AM and the last step is BER prediction.

### Dataset description

**Fig. 1:**
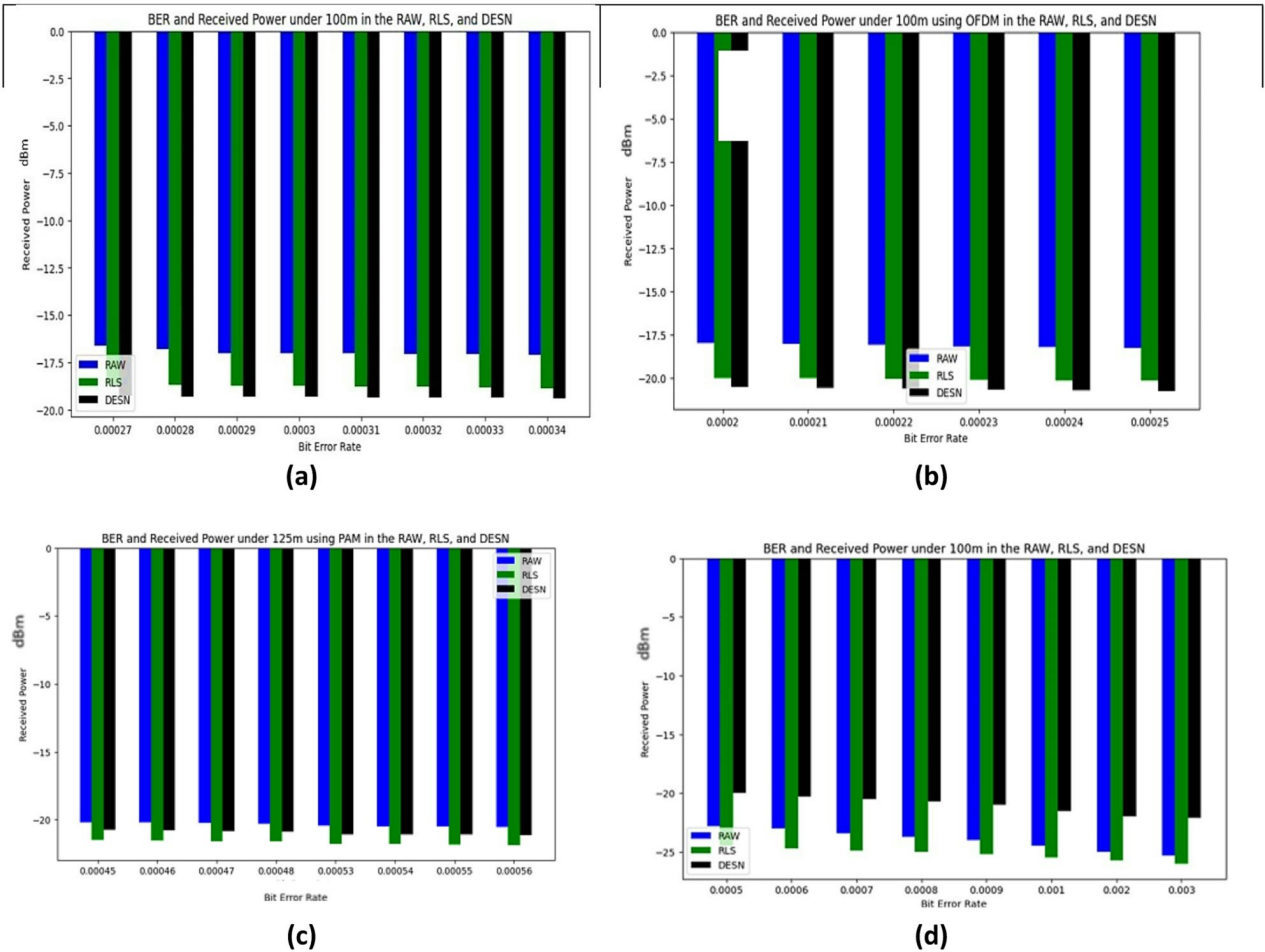
BER vs. received power under (**a**) 100 m in RAW, RLS, and DESN using QPSK-OFDM. (**b**) 110 m in RAW, RLS, and DESN using QPSK-OFDM. (**c**) 125 m in the RAW, RLS, and DESN using PAM. (**d**) 130 m in RAW, RLS, and DESN using PAM.

This paper uses DL models to perform this mission accurately. It should collect the number of data sets as input to enter the convolutional layer. The data sets refer to the values between the received power and the BER as recorded in^10^ using MATLAB. The data sets of BER indicate the range of errors in the received bits and their relation with the received power at the destination. Wang, K. et al. studied three underwater ranges such as 100, 125,167 m with different methods of channel estimation, RLS, and DESN. In our study, PAM is used to perform the modulation technique, where it is noticed that RAW and RLS achieve the best performance with less BER than DESN. Four underwater ranges (100, 110, 120, and 130 m) with three different methods of channel estimation (RAW, RLS, and DESN with QPSK-OFDM) are utilized to decrease the inter-symbol and intercarrier interferences. According to the recorded data, DESN achieved the best performance in the different ranges underwater. The datasets can be demonstrated as shown in the following figures. Figure 1a shows the relation between BER and received power under 100 m in the RAW, RLS, and DESN using PAM, while Fig. 1b demonstrates the relation between BER and received power under 125 m in the RAW, RLS, and DESN using PAM. Figure 1c, shows the relation between BER and received power under 100 m in the RAW, RLS, and DESN using QPSK-OFDM, and Fig. 1d displays the relation between BER and received power under 110 m in RAW, RLS, and DESN using QPSK-OFDM. To guarantee steady gradient behavior and uniform feature scaling across samples, all received power values were normalized to the range [0, 1] using min–max scaling prior to training. To guarantee statistical consistency, each model’s training and testing stages were carried out three times; the reported results show the average performance. Three subsets of the received power and related BER values dataset are randomly selected based on 70% for training, 15% for validation, and 15% for testing.

### Underwater optical wireless communication system

**Fig. 2:**
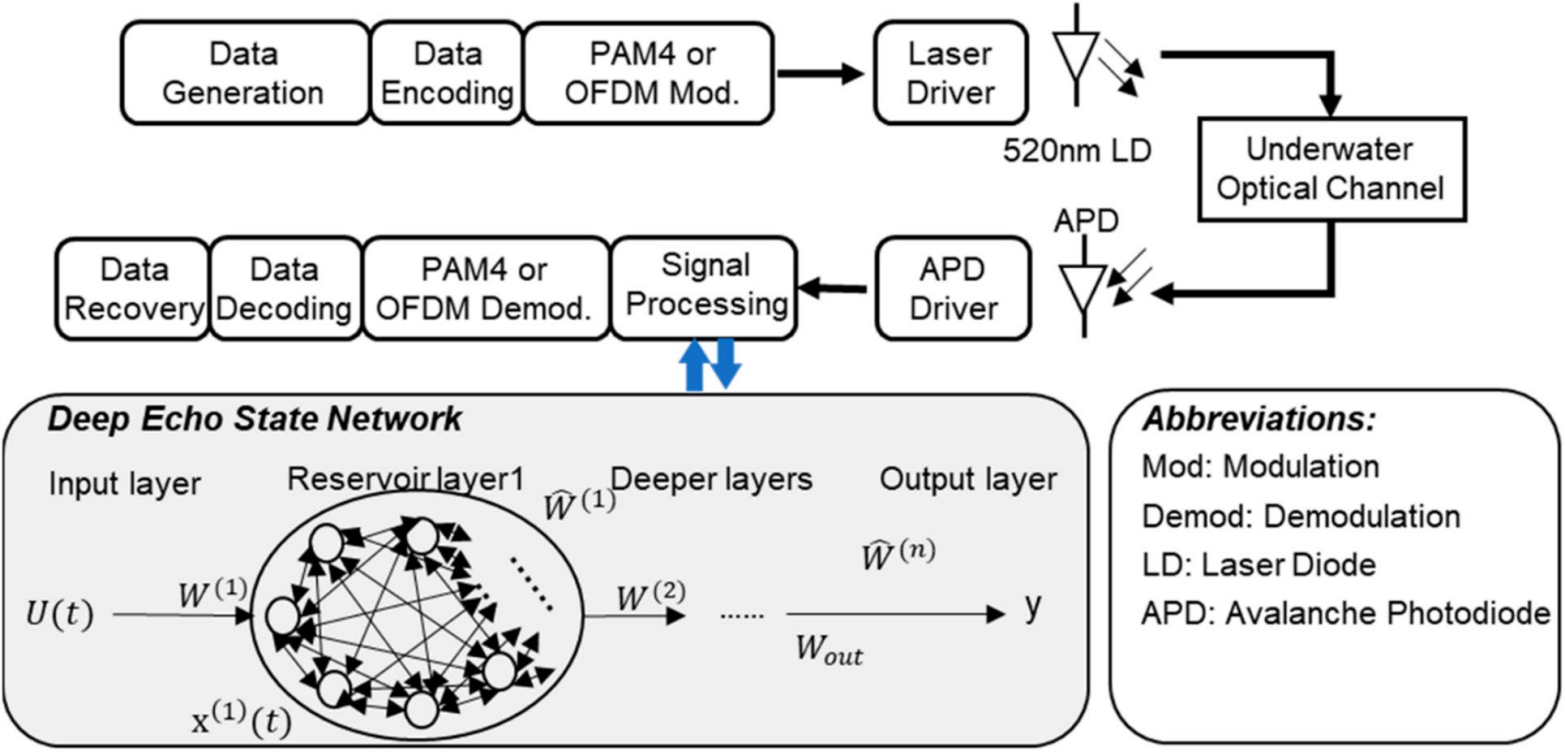
System configuration for DESN-assisted UWOC^10^.

The system configuration for DESN in a UWOC channel is demonstrated in (Fig. 2)^10^. The signal processing block represents the method used in channel estimation to recover the received bits. The signal processing block can be cancelled and is not used as in the RAW method; it also can be utilized by DESN or RLS. The PAM or QPSK-OFDM block refers to the type of modulation and demodulation techniques used in the system. The optical source used to transmit data is the Laser Diode (LD), while the receiver is an Avalanche Photodiode (APD).

### LSTM model

The Long Short-Term Memory (LSTM) capabilities are especially well-suited to process time-series data and sequences, which makes them ideal for UWOC systems. In our proposed framework, LSTMs are utilized to capture the temporal dependencies in UWOC signals. Additionally, LSTMs employ input gate, forget, and output gate to control the information flow, aiding in the retention of pertinent data and removing unnecessary data. Moreover, our datasets are normalized to a consistent range to improve training efficiency and performance.

### CNN model

The CNN model is composed of multiple convolutional layers which are the first in the CNN architecture for UWOC, then, followed by pooling layers and fully connected layers. The fully connected layers interpret the features to carry out operations like distortion correction and denoising. The convolutional layers extract features from the input datasets and the pooling layers reduce the dimensionality. Moreover, the activation function layer subjects the output of the first layer or the convolutional layer element by element. Additionally, the pool layer is in charge of reducing the volume and improving processing performance, both of which are input to the CNN with the main objective of avoiding any kind of overfitting.

The introduced CNN consists of three hidden layers with a learning rate of 0.002 which is adequate to guarantee that the model converges at a reasonable rate. Moreover, a 280 × 32 feature map is created by the first convolutional layer using 32 filters with a Kernel size of 3 × 3 and a stride of (1) Each feature map would be shrunk to 140 × 32 in the first pooling layer, a max-pooling layer with a pool size of 2 × 2 and a stride of (2) After the second hidden layer, convolutional and pooling, the output is 70 × 64 feature maps. Upon flattening, the output size is 4480 vector features. In that context, the batch size of 32 is reasonable to provide a good balance between training stability and computational efficiency and an easy model to integrate into the LSTM model.

Proposed CNN model used to be integrated with the RNN or LSTM model that CNN architecture uses two convolutional layers and pooling operations to gradually extract and condense features in order to analyze sequential data with an input shape of (280, 2). A pooling layer lowers the temporal dimension to 140 after the first convolutional layer increases the feature depth to 32 while preserving the sequence length. The sequence is further down sampled to a length of 70 by a subsequent pooling layer after a second convolutional layer raises the feature depth to 64. In order to feed the output into fully connected layers, it is finally flattened into a 1D vector of size 4480.

### CNN- RNN-AM model

In this work, temporal correlations in the input sequences are captured by modeling sequential dependencies in UOWC data using an RNN. To improve the performance of the BER prediction model for UOWC systems, the AM is a key component of the proposed study. Instead of considering all time steps equally, AM’s primary goal is to assist the model in concentrating on the most pertinent portions of the input sequence.

RNNs may have trouble recognizing long-term dependencies or determining which input elements are most instructive for the output prediction because they process sequences step-by-step. By using attention, the model generates a context vector, which is a weighted sum of all hidden states, and calculates attention weights for every time step in the sequence. For precise BER prediction, the fully connected layer receives this context vector, which condenses the most important details from the sequence. The mathematical equations are clarified as follows:

1 $$\:X=\{{x}_{1},{x}_{2},\dots\:\dots\:\dots\:\dots\:.{x}_{T}\}$$

2 $$\:{h}_{t}=RNN\:\left({x}_{t},\:{h}_{t-1}\right),\:\:t=\mathrm{1,2},\dots\:.T$$

where $\:X$, $\:{x}_{t}\in\:{R}^{d}$, $\:{h}_{t}\in\:{R}^{n}$, $\:T$ represents the input sequence, input at time step $\:t$, hidden state $\:{h}_{t}$ at time $\:t$, total number of time steps respectively.

The dataset includes a variety of underwater channel conditions and modulation methods, such as QPSK-OFDM and PMM. The AM layer provides richer contextual information by highlighting the most pertinent aspects throughout the time steps, while RNN layers process the input data, which is organized as vector sequences of length 20, to capture temporal dependencies.

To anticipate the BER, a fully connected layer receives a context vector from the attention-enhanced RNN outputs. In order to balance convergence speed and gradient stability, the model is trained using the Adam optimizer with a learning rate of 0.001, batch size of 64, and MSE as the loss function across 150 epochs. The convolutional layers also use a 2 × 2 Kernel size to extract fine-grained features, and a dropout rate of 0.2 reduces overfitting and improves generalization. This framework improves the resilience and forecast accuracy of BER under a variety of UOWC settings by efficiently utilizing CNN for spatial feature extraction and AM for concentrating on important temporal patterns.

Three convolutional layers with filter widths of 64, 64, and 128 are described in the model architecture with Kernel Size (4,4), Max-Pooling (3, 3) and 1 RNN layer. This enables efficient hierarchical feature extraction, especially from structured spatial inputs like signal matrices.

The model architecture that successfully blends attention-based refinement and spatial feature extraction can be described as the following, the addition of additive attention after the CNN layers improves the model’s emphasis on the most pertinent features by dynamically weighting significant regions, while the three convolutional layers with increasing filter depth (64, 64, and 128) enable progressive feature learning with additive attention after CNN layers and Global Max Pooling. By lowering dimensionality and maintaining dominating activations, Global Max Pooling reduces the feature map to a single vector. Lastly, binary classification tasks are a good fit for the dense output layer with a Sigmoid activation. All things considered, the design is simple yet effective, providing enhanced performance and interpretability through focus.

### The CNN with LSTM hybrid model

More flexible and adaptive solutions are desperately needed to improve the UOWC spectral utilization efficiency. The key to enhancing our proposed framework performance and reducing complexity is to minimize noise and distortion effects based on CNN-LSTM and improve system tolerance to low SNR in received signals from UWOC channels. In this study, the CNN-LSTM model is applied to the BER values and received power to overcome the overfitting and high complexity.

The procedure in our proposed model is as follows. Sequences of received power values and matching BER values made up the input data for CNN. There is a set length of 20 time steps for each sequence. Each sequence is processed by the CNN layer to extract and identify spatial features and local patterns in the input data. After being reshaped, the CNN-extracted features are sent to the LSTM layer for processing to extract sequential information and temporal dependencies. The final output generated by the LSTM layer is usually a prediction of the BER for the specified order of received power values.

There are two phases to our framework: an online phase and an offline phase. A dataset of received power with a known BER is used to train CNN (online phase). To reduce the error between the predicted outputs and the actual signals, the network’s weights are adjusted during the training process. After adjusting the CNN-LSTM model parameters, the testing phase is applied (offline phase). In this phase, the received power is used as input to our model to predict the BER. The proposed parameters are represented in (Table 1).

**Table 1:** Parameters of CNN-LSTM model.

Setup	CNN-LSTM
Learning rate	0.001
Optimizer	Adam
Activation function	Sigmoid
Kernel size	(4 × 4)
Batch size	32
Max-pool	(4 × 4)
Dropout	0.5
Stride	1
Height	32
Width	32
Epoch	100

### Integration of CNN with LSTM hybrid with AM

Lastly, a hybrid DL model can combine AM, LSTM, and CNN, utilizing each component’s strengths. The AMs are used to focus on the most important portions of the input, CNNs are used to extract spatial features, and LSTMs are used to extract temporal dependencies.

The hyperparameters of the hybrid DL model; CNN-LSTM-AM, that predicts the BER in UOWC systems by merging CNN, LSTM, and an AM is described in (Table 2). The CNN retrieves spatial features, the LSTM records temporal dependencies, and the AM highlights the most pertinent portions of the input sequence. All of which contribute to the robust model construction and improve contextual learning.

**Table 2:** Hyperparameters of the CNN-LSTM-AM.

Component	Description	Explanation
Model	AM	Focus on most important input
	CNN	Spatial feature extraction
	LSTM	Temporal dependency extraction
Training parameters	Learning rate	0.002
	Epochs	300
	Batch size	16
CNN configuration	Kernel size	3 × 3
	Dropout rate	0.3
LSTM configuration	Output dimensions	64 units
Attention mechanism	Number of attention heads	2
	Key and query dimensions	64 × 64 (match LSTM output)
Activation function	–	Sigmoid

A focus on deep, steady learning at the expense of longer training time is indicated by the modestly adjusted training parameters, which include a learning rate of 0.002, 300 epochs, and a batch size of 16. Fine-grained patterns can be effectively captured by the CNN Kernel size of 3 × 3, and overfitting can be minimized by a dropout rate of 0.3. Effective attention integration into temporal learning is made possible by the 64-unit LSTM layer and the 64-by-64 key-query AM with two attention heads.

In situations when the BER prediction is confined between 0 and 1, the Sigmoid activation function is appropriate. To comply with model input requirements, input power values are appropriately molded and transformed into a Data Frame. Although using quantitative measures like RMSE could provide more thorough insight, evaluation by charting expected versus actual BER offers visual validation. All things are considered; the setup has a finely calibrated architecture that can record temporal and spatial dynamics and provide contextual focus through attention, making it appropriate for intricate underwater communication settings.

The main target of utilizing AM in the CNN-LSTM model is thus improving the signal quality by decreasing noise and consequently decreasing the BER of the received signal.

### Integration of TCN with LSTM hybrid with AM

In order to improve noisy optical signals in UWOC systems, the suggested methodology combines TCN, LSTM, and an AM into a single DL framework. In order to extract multi-scale temporal features, the model starts with an input layer that receives time-domain sequences representing distorted optical signals. The TCN network can capture fine-grained temporal patterns since it has five TCN blocks with 64 filters and a Kernel size of three. The model can effectively broaden its receptive field without adding more parameters by employing increasing dilation rates (1, 2, 4, 8, 16), which captures both short- and long-term dependency. The final dense layer with a Sigmoid activation is appropriate for binary classification, and a dropout rate of 0.3 is used to minimize overfitting.

The well-structured approach for improving UWOC signals is shown by the layered architecture shown in (Table 3). The model starts with an input layer set up for one feature channel and 100-length sequential data. After that, it moves through five stacked TCN blocks with progressively higher dilation rates (1–16), which are essential for preserving the original sequence length since causal convolutions are used to capture hierarchical temporal connections. 64 filters and a tiny Kernel size of 3 are used in each block, balancing feature richness and computational efficiency. While residual connections aid in maintaining gradient flow across deep layers, regularization and robust training are ensured by the use of batch normalization, ReLU activation, and dropout at a rate of 0.2.

**Table 3:** Parameters of the suggested TCN-LSTM-AM model.

Layer	Type	Parameters	Output Shape
Input	Input layer	Sequence Length: 100, Channels: 1	(None, 100, 1)
TCN Block 1	Dilated Conv1D	Filters: 64, Kernel Size: 3, Dilation Rate: 1	(None, 100, 64)
TCN Block 2	Dilated Conv1D	Filters: 64, Kernel Size: 3, Dilation Rate: 2	(None, 100, 64)
TCN Block 3	Dilated Conv1D	Filters: 64, Kernel Size: 3, Dilation Rate: 4	(None, 100, 64)
TCN Block 4	Dilated Conv1D	Filters: 64, Kernel Size: 3, Dilation Rate: 8	(None, 100, 64)
TCN Block 5	Dilated Conv1D	Filters: 64, Kernel Size: 3, Dilation Rate: 16	(None, 100, 64)
	BatchNorm + ReLU + Dropout	Dropout: 0.2, Residual connections applied	(None, 100, 64)
Bidirectional LSTM	LSTM Layer	Units: 64 (per direction), Return Sequences: True	(None, 100, 128)
Attention mechanism	Dense + Softmax + Weighted Sum	Units: 1, Activation: tanh, Attention across time steps	(None, 128)
Fully connected	Dense layer	Units: 64, Activation: ReLU	(None, 64)
Output	Dense layer	Units: 1, Activation: Linear	(None, 1)

After that, the TCN output is sent to a 64-unit bidirectional LSTM layer, which records temporal dependencies in both the past and the future. By adaptively weighting significant time steps, the AM plays a crucial function in helping the model concentrate on the sequence’s most instructive segments. The final prediction is then produced by a fully linked dense layer with ReLU activation and an output layer with linear activation, making it appropriate for regression applications such as signal recovery. Overall, it makes sense for the model to use both sequential memory and temporal convolution to improve denoising and signal reconstruction in noisy UWOC channels.

For every experiment, a set number of 300 epochs is used, in accordance with the data shown in Table 2. An early stopping criterion based on validation loss is also used, with a patience value of 20 epochs, to guarantee convergence and avoid overfitting. This means that training was automatically stopped and the best model weights (with the lowest validation loss) were restored if the validation loss did not improve during 20 consecutive epochs.

### Hardware specifications

The Google Colab Pro + GPU environment is used to implement and run all DL experiments, including the CNN-LSTM, CNN-LSTM-AM, and TCN-LSTM-AM architectures:

- NVIDIA Tesla T4 and A100 GPUs, each with the following specs, are accessible through the platform.
- GPU: NVIDIA Tesla T4/A100 (CUDA-enabled, 16–40 GB GDDR6 VRAM) Version 12.1 of the CUDA Toolkit and Version 8.9 of cuDNN.
- RAM: DDR4, 12–25 GB.
- 100 GB of temporary disk space for storage.
- Python 3.10, TensorFlow 2.14.0, Keras 3.0, NumPy 1.26, and Matplotlib 3.9 comprise the software stack.

## Results and discussion

**Fig. 3:**
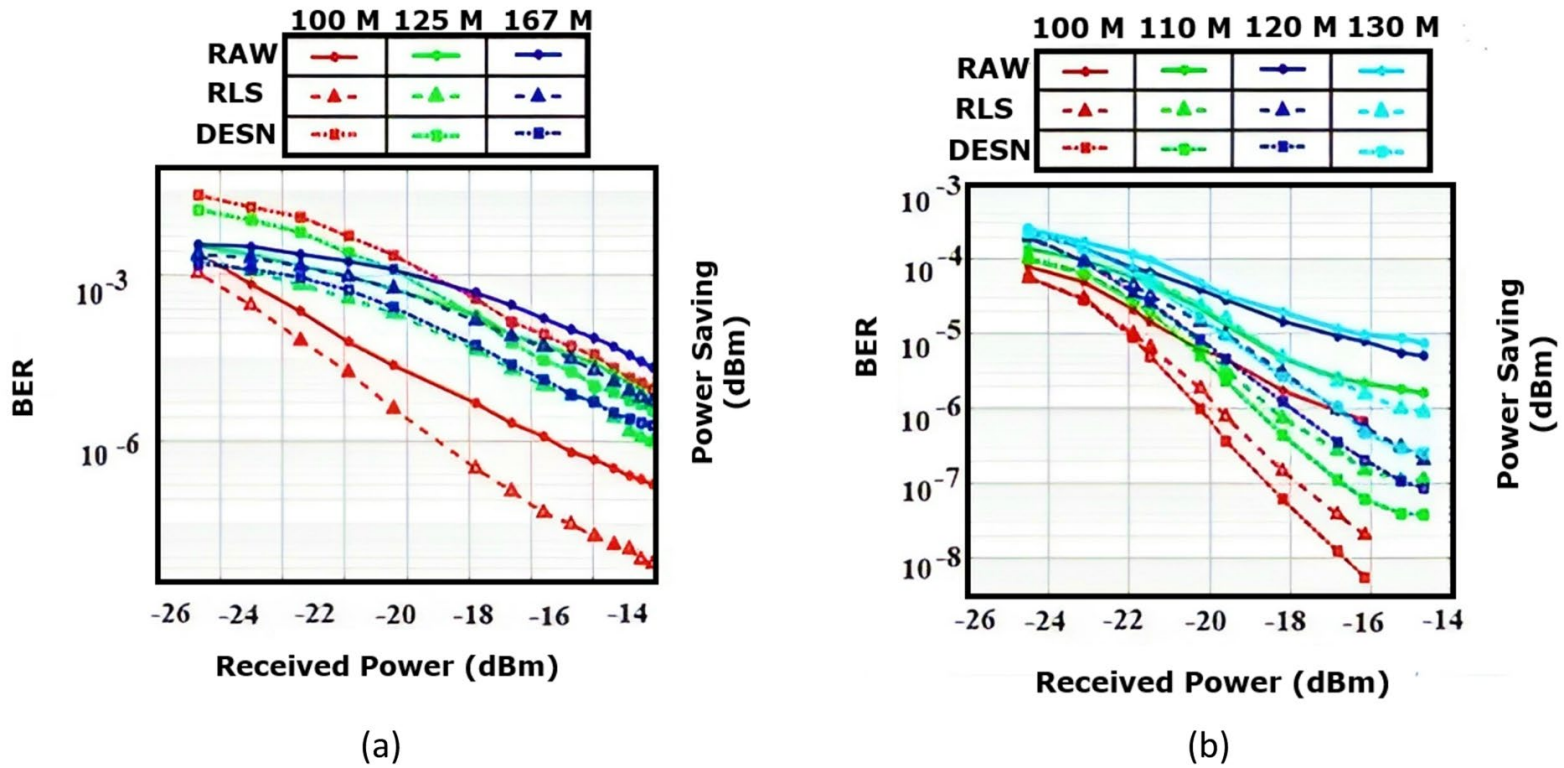
CNN-RNN-AM model on BER curves for (**a**) PAM and (**b**) QPSK-OFDM modulations with different transmission speeds and signal-processing methods (RAW for no equalization, RLS for recursive least squares filter, and DESN for DESN processing).

The simulation results and a discussion of the predetermined model are presented in the following figures. The results are compared and the performance is improved which means a decrease in the BER. Figure 3a uses PAM modulation for 100,125,167 m underwater and shows the comparison between three methods, RAW, RLS, and DESN.

The DESN achieves the best performance for 167 m, while RLS achieves the best performance for both 100, and 125 m, without any channel estimation signal processing and RAW makes the worst performance for all different ranges underwater 100, 125, and 167 m. By comparing the results with that in Ref^10^. , no improvement in BER is noticed.

**Fig. 4:**
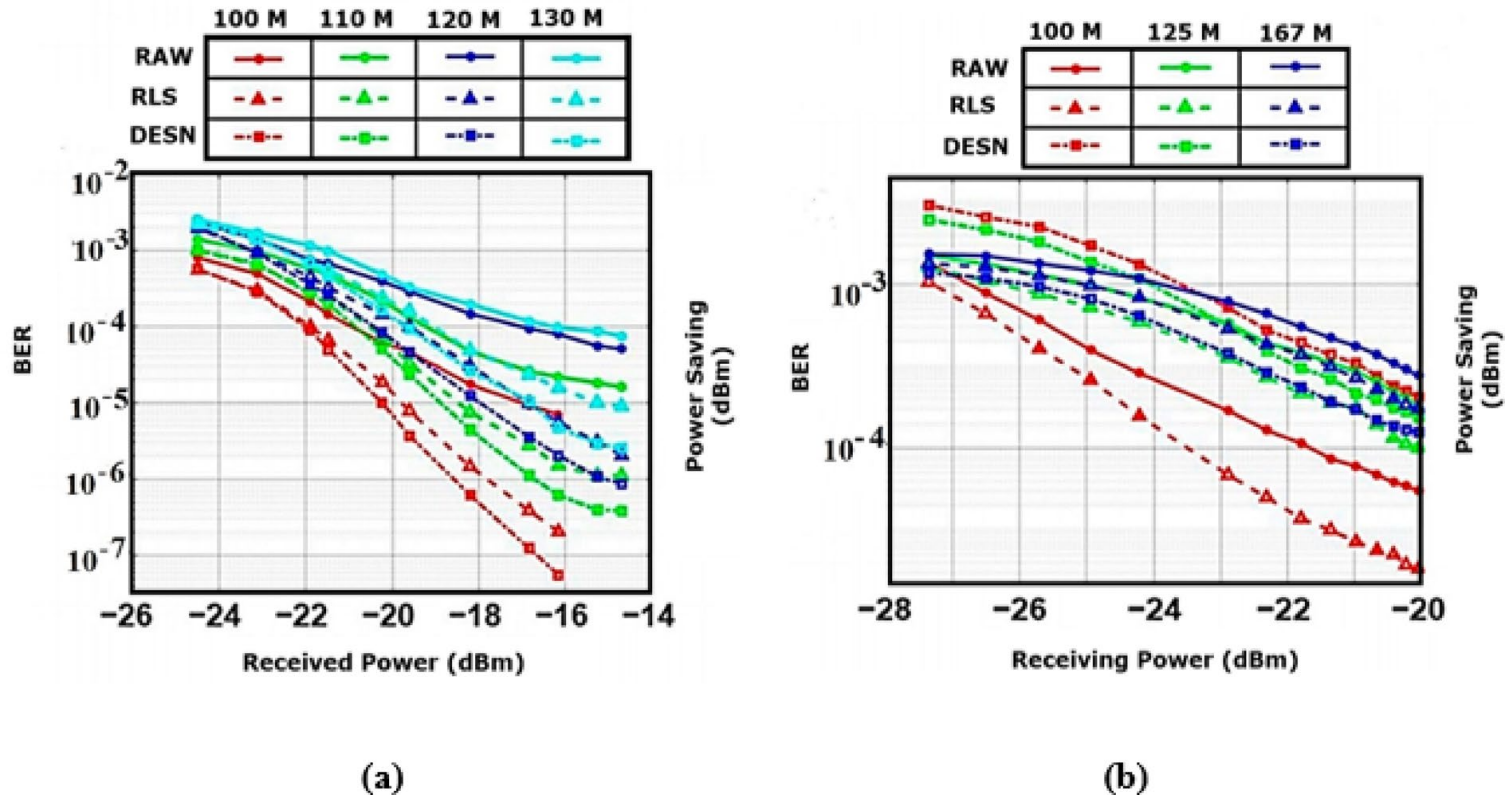
Applied CNN-LSTM-AM neural network model on BER curves for (**a**) PAM and (**b**) QPSK-OFDM modulations with different transmission speeds and signal-processing methods (RAW for no equalization, RLS for recursive least squares filter, and DESN for DESN.

Figure 3b uses QPSK-OFDM for 100,110,120,130 m and applies the CNN-RNN model using AM. The results in Fig. 4b for all ranges utilizing QPSK-OFDM are better than those in Fig. 3a in general. Also, DESN achieves the best performance with the lowest values of BER over that obtained by RLS, and RLS results are better than that by RAW. By comparing the results of Fig. 4b with those demonstrated in Ref^10^. , the improvement is summarized. The improvement percentage of using CNN-RNN-AM with QPSK-OFDM in this paper achieves nearly 18.9, 23.5, 33.3, and 29.4% for ranges 100, 110, 120, and 130 m, respectively.

Concerning the CNN-LSTM-AM, the relation between BER and received power is shown in Fig. 4 by utilizing PAM and QPSK-OFDM modulations for ranges 100,125, 167 m underwater using the techniques RAW, RLS, and DESN.

From the results, one can observe that, under both 100, and 125 m, RLS has the best performance with the least BER, while DESN under 167 m achieves lower BER and obtains the optimum results under the deepest range of 167 m. By comparing the results with that in Ref^10^. , one can conclude that using CNN-LSTM-AM improves the performance at the beginning of the curves then the results seem to be similar. At the beginning of the RLS curve of 100 m underwater, the improvement is 5.8%, and for the DESN curve is about 30.2% of 167 m underwater. In the center and end of curves, there is no improvement, which means, in general, the average percentage of the improvement of using CNN-LSTM-AM in PAM modulation is very tiny as shown in (Fig. 4).

**Fig. 5:**
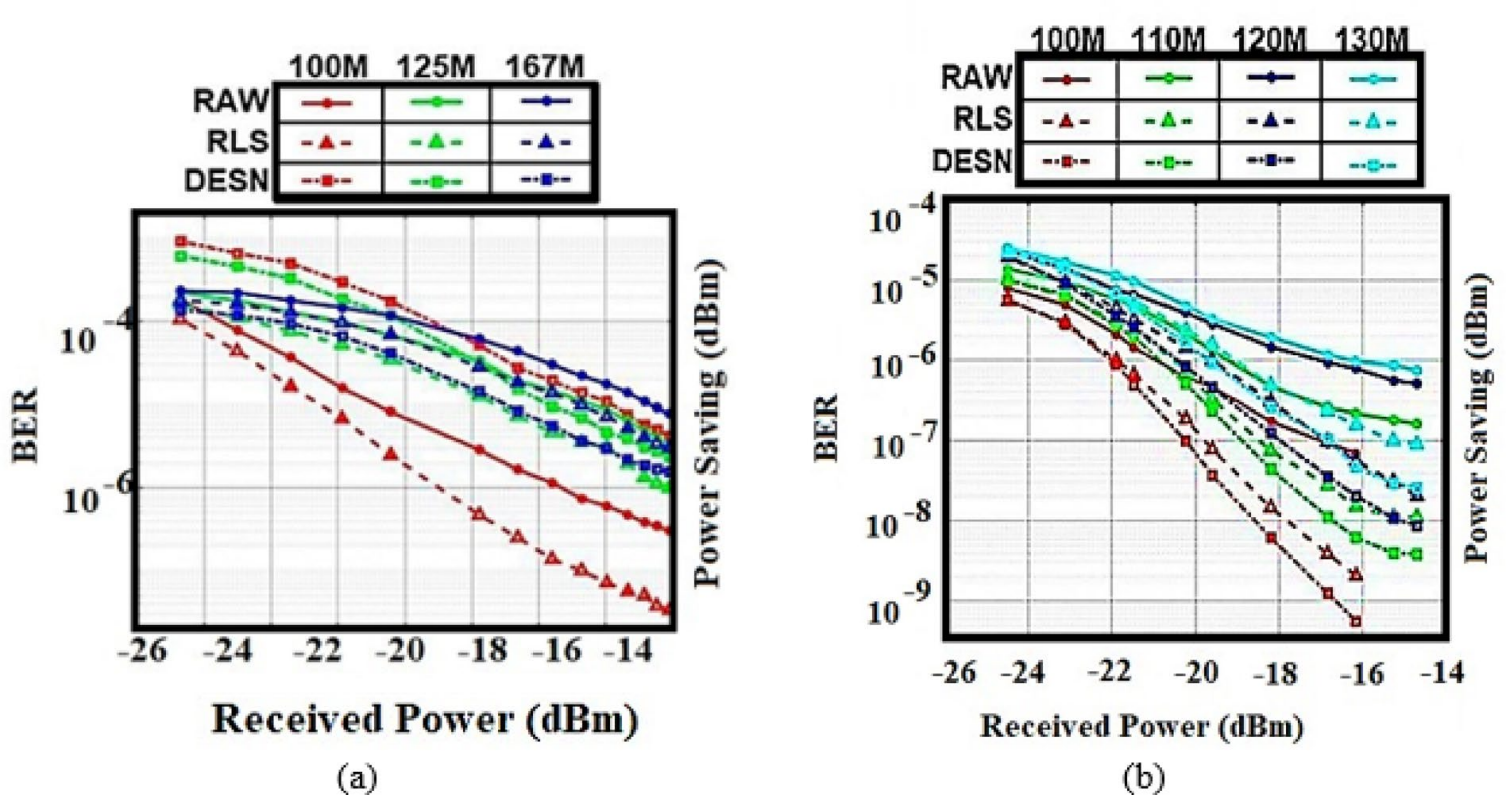
Applied TCN-LSTM-AM neural network model on BER curves for PAM (**a**) and QPSK-OFDM (**b**) modulations with different transmission speeds and signal-processing methods (RAW for no equalization, RLS for recursive least squares filter, and DESN for DESN processing).

On the other hand, using CNN-LSTM-AM with QPSK-OFDM is demonstrated in (Fig. 5b). for ranges 100, 110, 120, and 130 m underwater. DESN surpasses both RLS and RAW for all ranges and achieves the best performance. The comparison between Fig. 5(b) and Ref^10^. gets some improvement, where the improvement of using DESN with CNN-LSTM-AM and QPSK-OFDM related to the ranges are 10%,10.5%,11.11%, and 17.6%, respectively, for ranges 100, 110, 120, 130 m underwater.

Combining the techniques TCN-LSTM with AM to improve the overall performance by improving the signal quality by decreasing the noise and BER at the received signal is illustrated in Fig. 6, where (a) uses PAM and (b) uses QPSK-OFDM. Figure 6(a) illustrates the large effect of AM with the TCN-LSTM model for three methods RAW, RLS, and DESN.

From the results, RLS surpasses both DESN and RAW for 100, and 125 m underwater, while DESN achieves the best performance over RLS and DESN for 167 m underwater. RLS, in Fig. 4(a), is better than that in Ref^10^. by 17.3% for a range of 100 m underwater, while DESN, in Fig. 5(a), is better than that in Ref^10^. by 25% for the range 167 m underwater. QPSK-OFDM with DESN, RLS, and RAW methods of signal processing are combined with TCN-LSTM-AM, and the obtained results are shown in Fig. 5(b), where the combined techniques outstand other techniques. This achieves an overall best performance improvement by 29.4%, 41.17%, 53.3%, 71.4% for ranges 100, 110,120,130 m, respectively.

The influence of combining temporal convolutions, AM, and recurrent structures in improving prediction accuracy and decreasing error in BER estimation tasks is evident from the performance comparison of the listed models, as illustrated in Fig. 5. With a comparatively high MSE of 0.013, RMSE of 0.105, and MAE of 0.083, the baseline CNN model exhibits the lowest accuracy of 87.64% and limited capacity for temporal learning. CNN-LSTM-AM achieves a large accuracy boost of 96.77% and noticeably lower MSE of 0.0054 and MAE of 0.054, illustrating the usefulness of merging sequential memory and AM. The performance improves gradually across CNN-based models by incorporating RNN and AM. Similarly, because TCN-based models are better in managing temporal patterns without recurrent dependencies, they typically perform better than CNN variants.

As shown in Table 4, by combining TCN parallel temporal processing with LSTM long-term memory and the attention mechanism’s selective focus, the TCN-LSTM-AM model performs best overall, with the highest accuracy of 97.99%, lowest MSE of 0.0044, RMSE of 0.061, and MAE of 0.049. In order to achieve the best prediction accuracy in BER estimation, this development emphasizes the importance of deep hybrid designs that take advantage of both temporal and attention dynamics.

**Table 4:** Comparison between our proposed models.

Model	Accuracy (%)	MSE	RMSE	MAE
CNN	87.64	0.013	0.105	0.083
CNN-RNN	91.21	0.011	0.109	0.078
CNN-AM	92.65	0.010	0.100	0.079
CNN-RNN-AM	93.64	0.0078	0.084	0.078
CNN-LSTM	94.33	0.0064	0.083	0.079
CNN-LSTM-AM	**96.77**	**0.0054**	**0.073**	**0.054**
TCN	90.84	0.012	0.101	0.081
TCN-RNN	93.89	0.0109	0.104	0.071
TCN-AM	93.47	0.0087	0.077	0.069
TCN-RNN-AM	94.00	0.0078	0.071	0.087
TCN-LSTM	94.54	0.0061	0.072	0.061
TCN-LSTM-AM	**97.99**	**0.0044**	**0.061**	**0.049**

The findings unequivocally demonstrate how adding AM and TCN significantly improves the performance of signal enhancement models for UWOC. We consistently see gains in accuracy and error measures when comparing models with and without AM. When AM is added to CNN-LSTM, for example, accuracy achieves 2.44% improvement, while MSE, RMSE, and MAE decrease from 0.0064 to 0.0054, 0.083 to 0.073, and 0.079 to 0.054, respectively. In a similar, way, TCN-LSTM accuracy increases with 3.65% gain and shows significant error reductions (MSE decreases from 0.0061 to 0.0044).

Combining LSTM with AM greatly enhances model performance, as Table 5 demonstrates. TCN-LSTM-AM achieves the largest error reductions, MSE of 66.15%, RMSE of 41.90%, MAE of 40.96% and the highest accuracy improvement of 11.80%. CNN-LSTM-AM and other models demonstrate that combining attention and temporal modeling (LSTM) improves accuracy and lowers prediction errors in BER estimation tasks. The models that combine LSTM and attention regularly perform better than the others.

**Table 5:** Improvement percentage for our proposed models compared to the baseline CNN model.

Model	Accuracy (%)	MSE (%)	RMSE (%)	MAE (%)
CNN-RNN	+ 4.07	15.38	-3.81	6.02
CNN-AM	+ 5.71	23.08	4.76	4.82
CNN-RNN-AM	+ 6.84	40.00	20.00	6.02
CNN-LSTM	+ 7.63	50.77	20.95	4.82
CNN-LSTM-AM	**+ 10.41**	**58.46**	**30.48**	**34.94**
TCN	+ 3.65	7.69	3.81	2.41
TCN-RNN	+ 7.13	16.92	0.95	14.46
TCN-AM	+ 6.64	33.08	26.67	16.87
TCN-RNN-AM	+ 7.25	40.00	32.38	-4.82
TCN-LSTM	+ 7.87	53.08	31.43	26.51
TCN-LSTM-AM	**+ 11.80**	**66.15**	**41.90**	**40.96**

One can conclude that there is a large improvement overall in the system by using the QPSK-OFDM technique with DESN rather than using PAM modulation. The best results are achieved by the CNN-LSTM-AM model where one can obtain the best performance under the deepest distance underwater. Both RNN and CNN-LSTM achieve a very good improvement but CNN-RNN-AM outperforms CNN-LSTM due to using AM which increases the quality of the signal and reduces noises and minimizes the BER at the received sign. Table 6 illustrates the improvement percentages when using the QPSK-OFDM technique for three DL models CNN-RNN-AM, CNN-LSTM, and CNN-LSTM-AM compared with the results obtained in Ref^10^.

**Table 6:** Improvement percentages when using different methods compared with the results in Ref^10^.

Modulation type	Depth underwater	CNN-RNN-AM	CNN-LSTM	TCN-LSTM-AM
QPSK-OFDM	100 m	18.9%	10%	29.4%
	110 m	23.5%,	10.5%	41.17%
	120 m	33.3%	11.11%	53.3%
	130 m	29.4%	17.6%	71.4%

Now, one can present a concise synthesis of all key findings, where, TCN-LSTM-AM achieves the largest error reductions, MSE of 0.0044%, RMSE of 0.061%, MAE of 0.049% and the highest accuracy improvement of 97.99%. The combined techniques outstand other techniques. This achieves an overall best performance improvement by 29.4%, 41.17%, 53.3%, 71.4% for ranges 100, 110,120,130 m, respectively.

## Conclusion

In this paper, the main strength is represented in the enhancement of UWOC system performance for both PAM and QPSK-OFDM techniques. The channel estimation by signal processing DESN outperforms that obtained by RLS and RAW in performance. PAM with the CNN-RNN-AM model does not achieve any improvement with comparing the results in Ref^10^. QPSK-OFDM combined with CNN-RNN-AM and DESN gets good results and the improvement of nearly 18.9%, 23.5%, 33.3%, and 29.4% for ranges 100, 110, 120, and 130 m, respectively. CNN-LSTM using PAM modulation outperforms TCN-RNN-AM. CNN-LSTM with QPSK-OFDM achieves some improvement using DESN, where we record the percentage improvement of about 10%, 10.5%, 11.11%, and 17.6%, respectively of ranges 100, 110, 120, 130 m underwater. The best performance, in this paper, is observed in the combination of the TCN-LSTM-AM model for QPSK-OFDM with using DESN which reaches the highest improvement and achieves improvement percentages by about 29.4%, 41.17%, 53.3%, and 71.4% for ranges of 100, 110,120,130 m underwater over RLS and RAW.

When comparing models with and without AM, we regularly observe improvements in accuracy and error metrics. For instance, an accuracy of 2.44% improvement when AM is added to CNN-LSTM, whilst MSE, RMSE, and MAE fall from 0.0064 to 0.0054, 0.083 to 0.073, and 0.079 to 0.054. Similar to this, TCN-LSTM accuracy exhibits notable mistake reductions, MSE drops from 0.0061 to 0.0044, and grows with attention gain of 3.65% gain.

The strongest performance is observed in our proposed TCN with LSTM framework, as it captures the strong spatial patterns in the optical signals in the dataset before processing the temporal aspects. For LSTM to model temporal dependencies, TCN must first extract spatial features. The TCN-LSTM-AM model increases the communication system dependability and resilience in challenging underwater environments.

The future work includes performance enhancement by using different models of neural networks. One can also try to perform a combining system in less computational methods. A similar scenario could be created for the proposed work by considering the different conditions of the water as particles, sunlight, and diffraction cases.

## Data Availability

The data used and/or analyzed during the current study are available from the corresponding author on reasonable request.
